# The Christian Buddha

**Published:** 1890-01

**Authors:** 


### THE CHRISTIAN BUDDHA.

The late J. Crossett, the independent American missionary, is described in an official communication to the State Department at Washington, in the highest terms, as devoted to doing good to the poor: "He was known by the Chinese as the 'Christian Buddha.' He was attached to no organization of men. He was a missionary, pure and simple, devoted to charity rather than proselytism. He literally took Christ as his exemplar. Innkeepers would take no pay from him, and were ever glad to entertain him. It must be said that his wants were few He wore the Chinese dress, had no regular meals, drank only water, and lived on fruit, with a little rice or millet. He aimed at translating his ideal, 'Christ into reality.' He found good in all religions. After a long conversation with him one day, I told him he was not a Christian, but a Buddhist. He answered that there were many good things in Buddhism. He completely sacrificed himself to the good of the poorest of the poor. He acted out his principles to the end. He was poor and lived as plainly as the poorest of his patients. On charitable subjects he wrote well. The ideal to him was practical. Let this American be enshrined in the annals of men who loved their fellow men."

